# Prognostic Significance of Tissue Inhibitor of Metalloproteinase-1 in Breast Cancer

**DOI:** 10.1155/2012/290854

**Published:** 2012-09-04

**Authors:** Arunee Dechaphunkul, Monlika Phukaoloun, Kanet Kanjanapradit, Kathryn Graham, Sunita Ghosh, Cheryl Santos, John R. Mackey

**Affiliations:** ^1^Department of Oncology, Cross Cancer Institute, Faculty of Medicine & Dentistry, University of Alberta, Edmonton, AB, Canada T6G 1Z2; ^2^Holistic Center for Cancer study and Care (HOCC-PSU) and Division of Medical Oncology, Department of Internal Medicine, Prince of Songkla University, Songkhla, Thailand; ^3^Department of Pathology, Faculty of Medicine, Prince of Songkla University, Songkhla, Thailand

## Abstract

*Introduction*. Despite advances in breast cancer systemic treatment, new prognostic and predictive factors are still needed. Tissue inhibitor of metalloproteinase-1 (TIMP-1), a physiologic inhibitor of matrix metalloproteinases (MMPs), can act in both pro- and antitumoral effects. As role of TIMP-1 in breast cancer is controversial, we aimed to determine the prognostic significance of TIMP-1 in breast cancer. *Methods*. A single center-based case-control study was applied. Primary breast cancers from women with early stage disease treated with standard adjuvant therapy were analyzed by gene expression microarrays and immunohistochemistry for TIMP-1. *Results*. At the optimized cut-point, patients with high TIMP-1 RNA levels had a significantly shorter time to relapse, with a hazard ratio (HR) of 1.64 (*P* = 0.04), but without significant differences in overall survival (HR 1.29, *P* = 0.37). Although cytoplasmic overexpression of TIMP-1 protein was not correlated with early relapse (HR 1.0, *P* = 0.92), there was a tendency for short overall survival in patients with high expression (HR 1.41, *P* = 0.21). *Conclusions*. Our data indicate that elevated TIMP-1 RNA levels are independently prognostic for early recurrence, and there is a tendency for association of high cytoplasmic TIMP-1 protein levels with short survival in primary breast cancer.

## 1. Es fIntroduction

 Breast cancer is the second most common cause of cancer death in women, with more than 1 million new cases of breast cancer diagnosed each year [1]. In 2011, more than 23,000 Canadian women were newly diagnosed with breast cancer and 5,100 women died from this disease [2].

 Breast cancer is a heterogeneous disease characterized by varying morphological appearances, molecular features, and response to therapy [3]. Adjuvant systemic therapy in women with early stage disease is guided by prognostic and predictive factors, including stage, grade, estrogen receptor (ER) and progesterone receptor (PR) status, and HER2 amplification. These parameters help physicians to select adjuvant systemic therapy. However, these remain imperfect tools, in that some patients receive systemic chemotherapy even though they can be cured by surgery alone. In contrast, those who were categorized in low-risk group had short disease-free survival without receiving adjuvant chemotherapy. Therefore, new prognostic and predictive factors are still required to optimize treatments among these patients. 

 Tissue inhibitor of metalloproteinases-1 (TIMP-1) is one of four natural inhibitors of the matrix metalloproteinases (MMPs), the proteolytic enzymes responsible for degradation of extracellular matrix (ECM) and require for cancer dissemination. TIMP-1 is a multifunctional protein. In addition to its MMP-inhibitory function, it is also known to promote cell growth, inhibit apoptosis, and is probably involved in regulation of angiogenesis [4, 5]. Elevated levels of TIMP-1 mRNA and TIMP-1 protein have been found in many types of cancer, including breast cancer. Several studies reported the association between high levels of TIMP-1 and poor prognosis both at the mRNA and protein level in breast cancer [6–12]. Furthermore, some studies also reported TIMP-1 to be a predictive marker for chemotherapy and hormonal therapy, in which lack of response has been demonstrated in those with high TIMP-1 level [13, 14]. However, two studies have shown discordant results [15, 16]. Thus, the prognostic significance of TIMP-1 in breast cancer remains controversial.

 The objective of this study was to determine the prognostic significance of TIMP-1 RNA levels and cytoplasmic overexpression of TIMP-1 protein in a well-characterized and uniformly treated cohort of women with early stage breast cancer. 

## 2. Methods

### 2.1. Patient Selection

A single center-based case-control study was applied. One hundred and seventy-six biopsies from women with newly diagnosed early stage breast cancer obtained from the Canadian Breast Cancer Foundation Tumor Bank (CBCF TB) were analyzed by gene expression microarrays and immunohistochemistry (IHC) for TIMP-1.

 All patients underwent surgery, followed by standardized guideline-based adjuvant chemo- and/or hormonal therapies. With respect to chemotherapy, anthracycline-based regimens were recommended in patients with high risk node-negative disease whereas anthracycline with taxane chemotherapy was recommended in those with node-positive disease. All patients with ER-positive and HER-2 positive status received hormonal therapy and trastuzumab, respectively. Half of women in the study relapsed early (less than 5 years after the initial therapy), while half, matched for ER, HER-2 status, stage, age, and duration of followup, remained recurrence free. Three of the latter subsequently relapsed within five years. The median time of followup of surviving patients is 50.5 months.

### 2.2. Gene Expression Analysis

 The Agilent microarray platform was used for gene expression analysis as previously described [17]. The data was normalized using GeneSpring GX 7.3 (Agilent). Using the training data set from GEO Accession number GSE29210, the expression of TIMP-1 was assessed using a Kaplan-Meier curve analysis.

### 2.3. Tissue Microarray (TMA) Construction and Immunohistochemical Analysis

Formalin-fixed paraffin-embedded blocks were obtained from all patients in the study. Three 0.6 or 1.0 mm cores from each samples were constructed into a TMA using a TMArrayer (Pathology Devices, Westminster, Maryland) or a Beecher ATA-27 (Beecher Instruments. Inc, Sun Prairie, Wisconsin). TMAs were deparaffinized in xylene, rehydrated and microwaved for 20 min in epitope retrieval buffer (10 mM citrate, pH 6). TMAs were immunostained with anti-TIMP-1 antibody (1 : 100, Santa Cruz Biotechnology, Santa Cruz, CA).

### 2.4. Scoring and Quantification of Immunohistochemical Staining

TIMP-1 protein was scored for cytoplasmic localization. All immunostained slides were defined independently by two board certified pathologists blinded to clinical outcomes using the semiquantitative analysis based on the average staining intensity and relative abundance of immunoreactive cells throughout the tumor tissue. Specifically, intensity was graded on a scale of 0–3 ((0) no or barely detectable signal; (1) weak signal; (2) intermediate signal; (3) strong signal), whereas the relative abundance of immunoreactive cells was scored on a scale of 0–3 based on the percentage of epithelial cells stained positive ((0) less than 10%; (1) 10–50%; (2) 51–75%; (3) more than 75%). The sections were ultimately scored by combining the intensity and relative abundance of immunoreactive cells results (from 0 to 6) into the final scale of 0–3^+^ (score 0–0; score 1-2–1^+^; score 3-4–2^+^; score 5-6–3^+^). The scoring concordance between the two pathologists was excellent, with a weighted kappa value of 0.82 (95% CI: 0.74–0.91).

### 2.5. Statistical Analysis

 TIMP-1 immunohistochemical scoring was reported using frequencies and percentages. Weighted kappa statistics were used to test the degree of agreement between the scoring used by two pathologists. Overall survival was defined as the date of histologic diagnosis to the date of death; all living patients were censored at the last date of followup. Relapse-free survival was calculated as the date of diagnosis to the date of death or date of disease relapse. Again, the patients who were alive and did not experience recurrence were censored at the date of last followup. Kaplan-Meier curves were generated for overall survival and relapse-free survival. Kaplan-Meier curves were also generated for overall survival and relapse-free survival stratified by TIMP-1 final scores (0, 1^+^, 2^+^, and 3^+^) and log rank statistics was used to compare the curves. Cox's proportional hazard model was used to determine the hazard ratio and the corresponding 95% confidence interval. A significance level of 0.05 was used for all statistical analysis and two-sided tests were used. SAS software (SAS Institute Inc., Cary, NC, USA) version 9.1.3 was used for all statistical analysis.

## 3. Results

### 3.1. Prognostic Significance of TIMP-1 RNA Expression Levels

 A single center-based case-control study was applied. Gene expression microarrays were hybridized using RNAs isolated from samples obtained from 176 primary treatment-naive breast cancer patients (44 stage I, 118 stage IIA/IIB, and 14 stage IIIA/IIIB). Half of the patients developed early relapse (less than 5 years after the initial therapy), and half of them with matched ER, HER-2 status, stage, and age did not. The database for clinical outcomes was locked on April 22, 2011, at which time the median duration of followup was 50.5 months (range, 2.9–108.3). At the time of analysis, 91 of 176 patients had disease recurrence and 68 patients were dead (Table 1). 

 Gene expression analysis demonstrated relative TIMP-1 RNA levels in the 176 samples ranged from −5.80 to 4.38. A relative RNA level of 0.84 was the optimized cutoff point for the minimum *P* value associated with early recurrence. Among 176 patients, 38 (21.6%) had relative TIMP-1 RNA levels of ≥0.84. Univariate cox regression analysis showed a significant correlation to both recurrence and death for negative ER status, and high grade (defined as grade 3). Significant correlation of elevated TIMP-1 RNA levels and recurrence was also observed. However, there was no correlation to death or recurrence for HER-2 status and stage.

 High TIMP-1 RNA levels were found to correlate with HER-2 amplification. There was no correlation between relative TIMP-1 RNA levels of ≥0.84 and recurrence, death, negative ER status, and stage (Table 2).

**Figure 1:**
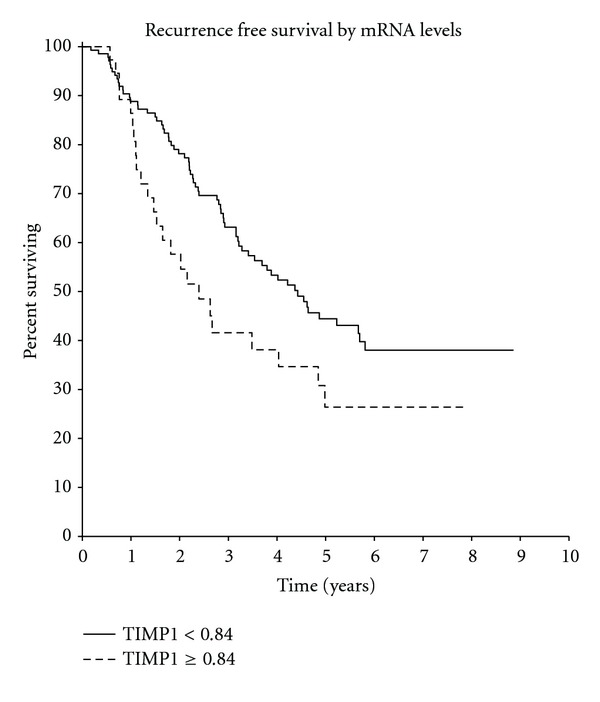
(HR = 1.64  *P* = 0.04).

**Figure 2:**
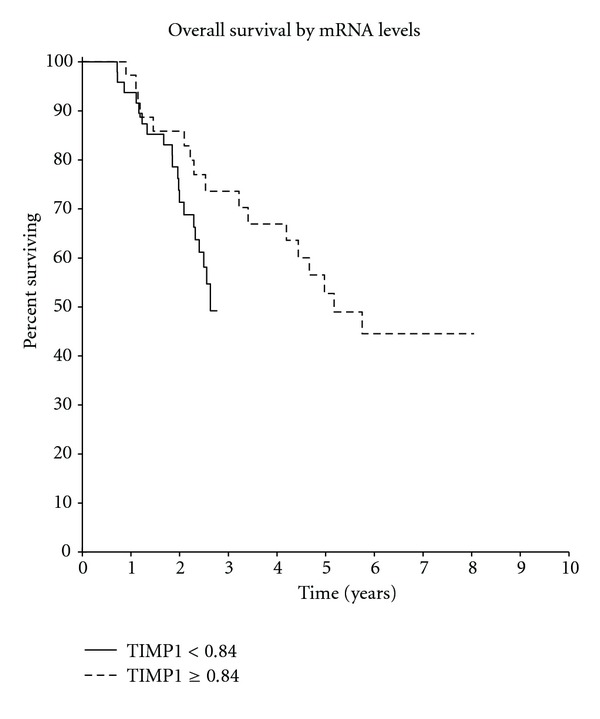
(HR = 1.29  *P* = 0.37).

Kaplan-Meier analysis showed that patients with high TIMP-1 RNA levels had a significantly shorter time to relapse, with a hazard ratio (HR) of 1.64 (*P* = 0.04) (Figure 1), but without significant differences in overall survival (HR 1.29, *P* = 0.37) (Figure 2). In multivariate analysis, when considering stage, histologic grade, hormonal, and HER2 status, TIMP-1 RNA levels remained independently prognostic for early relapse (HR 1.68, *P* = 0.04). There was no significant prognostic of the other covariates input into the multivariate analysis (Table 3).

### 3.2. Prognostic Significance of TIMP-1 Protein

**Figure 3:**
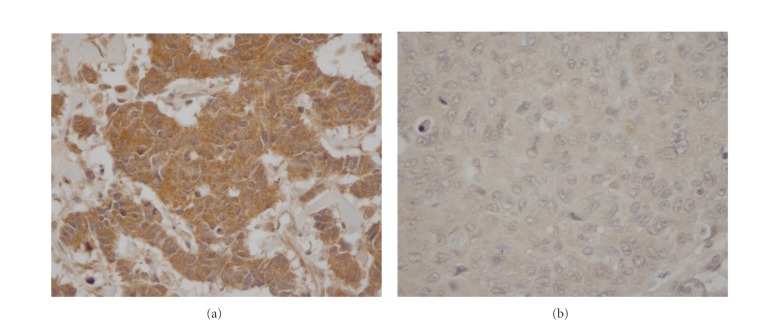
(a) High cytoplasmic overexpression of TIMP-1 (3^+^). (b) No cytoplasmic expression TIMP-1 (0).

 A TMA was generated using breast cancer tissue samples from all 176 patients, of which 31 were discarded due to insufficient tissue for scoring. Anti-TIMP-1 antibody was used for immunostaining on the TMA. TIMP-1 protein was primarily localized to the cytoplasm (Figure 3). Seventy-eight of the 145 patients represented in the TMAs recurred at the time of analysis, and 61 patients died (Table 4). High cytoplasmic abundance of TIMP-1 (scale 3^+^) was demonstrated in 94 of 145 breast cancer specimens (65%). 

High cytoplasmic TIMP-1 expression was not found to correlate with recurrence, death, negative ER status, HER-2 amplification, grade, or stage (Table 5). 

**Figure 4:**
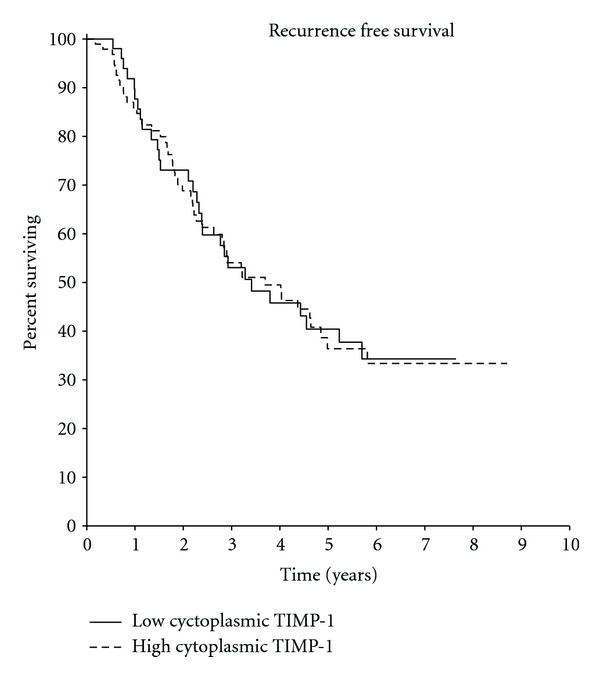
(HR = 1.0  *P* = 0.92).

**Figure 5:**
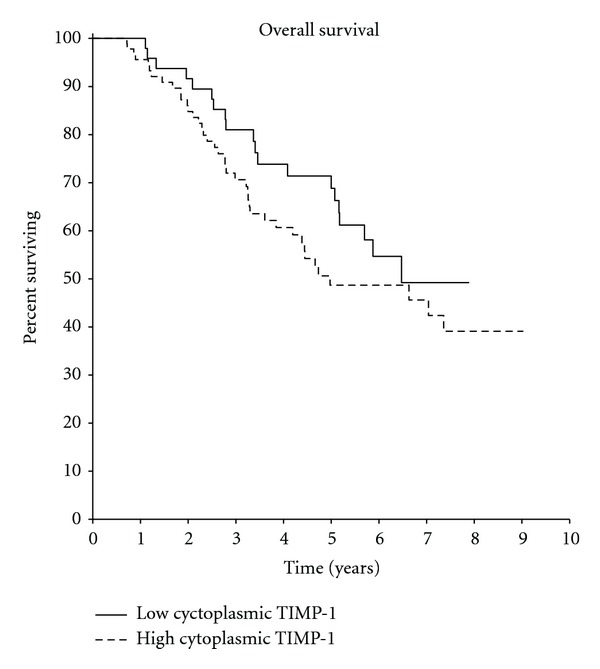
(HR = 1.41  *P* = 0.21).

In the univariate analysis of the 145 samples, although cytoplasmic overexpression of TIMP-1 protein was not correlated with early relapse (HR 1.0, *P* = 0.92), there was a tendency for short overall survival in patients with high expression (HR 1.41, *P* = 0.21; Figures 4 and 5). 

## 4. Discussion

 Curative-intent therapy in early stage breast cancer remains challenging, largely due to a growing appreciation of the molecular heterogeneity of the disease. Despite advances of systemic therapy in breast cancer guided by hormonal status and HER2 amplification, new prognostic, and predictive factors are still needed to optimize treatments among these patients. 

 In our study, we determined the prognostic significance of TIMP-1 RNA expression and protein abundance using gene expression and immunohistochemical (IHC) analysis of the primary tumors from 176 treatment naïve, early stage breast cancer patients. We found a significant correlation between high TIMP-1 RNA expression and early relapse, with a hazard ratio (HR) of 1.64. In multivariate analysis, TIMP-1 RNA levels remained independently prognostic for early relapse (HR 1.68); this result demonstrated that TIMP-1 provided prognostic information beyond stage, histologic grade, hormonal, and HER2 status. There was also nonsignificant association between overall survival and TIMP-1 RNA levels (HR 1.29, *P* = 0.37). Although quantitative RT-PCR has not been performed for this study, similar studies using the same platform demonstrate a high degree of correlation between gene expression microarray data and RT-PCR [18] and quantitative RT-PCR (KG, unpublished results).

 The incidence of TIMP-1 protein overexpression in our study (65%) is slightly lower than a previous report of 73%, although we used similar antibody and semiquantitative scoring criteria [6]. This may reflect differences in clinicopathological parameters and molecular subtypes of breast cancer between the studies. We also found a tendency for association of high cytoplasmic expression of TIMP-1 with shorter overall survival (HR 1.41, *P* = 0.21). These results confirm the independent prognostic value of TIMP-1 in early stage breast cancer patients receiving adjuvant therapy. 

 Several studies reported the association between high levels of TIMP-1 and poor prognosis both at the mRNA and protein level in breast cancer (Table 6). In the largest study to date, Schrohl et al., showed high levels of TIMP-1 protein in tumor tissue cytosolic extracts were associated with short recurrence-free and overall survival in nearly 3,000 patients [10]. Wu et al. demonstrated poor recurrence-free and overall survival in patients with high levels of TIMP-1 protein and mRNA in paraffin-embedded tissue [6]. Nakopoulou et al. reported the association of high TIMP-1 mRNA expression determined by in situ hybridization with poor prognosis in paraffin-embedded tumor tissue [19]. However, the published literature includes two studies showing the opposite results. Nakopoulou et al. reported the favorable prognostic impact of TIMP-1 protein overexpression in breast cancer using IHC analysis [15]. Another study reported by Sieuwerts et al. showing the low levels of TIMP-1 mRNA, determined by quantitative reverse transcriptase-polymerase chain reaction (RT-PCR), carried a poor prognosis [16]. These discordant results evaluating the prognostic significance of TIMP-1 mRNA and protein in breast cancer might arise from the differences of method used for mRNA expression analysis, as well as the differences of antibodies and semiquantitative scoring criteria between the studies, or the play of chance. In addition to its prognostic value, TIMP-1 is also reported to predict response to treatment. Schrohl et al. observed the association between high tumor tissue levels of TIMP-1 protein and poor response to chemotherapy in patients with metastatic disease [13]. Furthermore, high serum TIMP-1 levels had significantly reduced response to letrozole and tamoxifen in metastatic breast cancer [14]. In patients with early stage disease, absence of TIMP-1 expression was shown to be associated with incremental benefit from anthracycline-containing chemotherapy based upon the data from two randomized studies (DBCG 89D and MA.5) [20–22]. 

 Although measurement of serum TIMP-1 was not performed in our study, there were several studies reported the significant correlation between high serum TIMP-1 levels and poor relapse-free survival [8, 11, 21]. Furthermore, one study also showed that serum levels of TIMP-1 correlated with tissue levels [6].

 TIMP-1 expression has also been reported to be associated with adverse pathologic features and poor prognostic factors in different types of cancer, including nonsmall cell lung cancer, colorectal cancer, gastric cancer, and childhood acute lymphoblastic leukemia [23–30]. 

 TIMP-1 is one of four natural inhibitors of the matrix metalloproteinases (MMPs), the proteolytic enzymes that play an important role in cancer dissemination. In addition to its MMP-inhibitory function, it can also promote tumoral effects by a number of mechanisms [4, 5]. Firstly, several studies indicated that TIMP-1 can stimulate cancer proliferation independent on its MMP-inhibitory function. The proposed mechanism is TIMP-1 has a direct interaction with an as yet unidentified cell receptor that stimulates intracellular signaling promoting proliferation of cells [31]. However, the intracellular pathway initiated by TIMP-1 was still debated. Yamashita et al. demonstrated a crucial role of tyrosine kinase in the signal transduction of TIMP-1 and also found that mitogen-activated protein (MAP) kinases plays a role in TIMP-1-dependent growth signaling [32]. Wang et al. found TIMP-1 activated the Ras pathway in osteosarcoma cell lines [33]. Secondary, TIMP-1 was well established as an inhibitor of apoptosis. TIMP-1 has been shown to bind to a cell surface protein complex, including CD63 and beta 1 integrin [34], which then induces intracellular signaling cascades through phosphorylation of PI-3 kinase, activating the Akt survival pathway [35]. Li et al. demonstrated antiapoptotic activity of TIMP-1 in breast cancer cell lines independently of its ability to stabilize cell-matrix interactions. In addition, TIMP-1 overexpression is also associated with activation of focal adhesion kinase (FAK) which is known to be related in cell survival pathway [36]. Finally, TIMP-1 is probably involved in regulation of angiogenesis, although this role remains controversial [4]. 

 TIMPs are secreted proteins, and it has been debated what kind of cells secrete TIMP-1 due to some reports showed TIMP-1 expression in the cancer cells, and some found TIMP-1 expression in the stromal components of the tumor. Recent report by Kuvaja et al. showed TIMP-1 produced by mesenchymal stem cells mimicking the stromal components of the tumor, not produced primarily by breast cancer cell lines. However, it was suggested that breast cancer cells can take up TIMP-1 produced by stromal cells, and thus displayed positive TIMP-1 immunoreactivity [37].

 In conclusion, we show that high TIMP-1 RNA levels independently correlated with early relapse in breast cancer regardless of stage, grade, hormonal, and HER2 status, whereas high cytoplasmic expression of TIMP-1 protein was associated with shorter overall survival. Whether TIMP-1 levels predict treatment outcomes requires confirmation in larger studies with homogeneous treatment determined by randomization. Furthermore, TIMP-1 appears to warrant evaluation as therapeutic target in breast cancer.

## 5. Conclusions

 We conclude from this study that high TIMP-1 RNA levels are an independent prognostic factor for early relapse, and there is a tendency for association of high cytoplasmic TIMP-1 protein levels with short survival. Therefore, these results support evaluation of TIMP-1 as a therapeutic target in breast cancer.

## Figures and Tables

**Table 1:** Clinicopathologic features of the patients included in the gene expression microarray analysis.

		Univariate analysis					
		Recurrence			Death		
		HR	95% CI	*P*	HR	95% CI	*P*
Number of patients	176						
Age at diagnosis							
Median	52 years						
Range	26–89 years						
Recurrence							
Events	91 (52%)						
Death							
Events	68 (39%)						
ER status							
Negative	64 (36%)	1.54	1.00–2.38	0.05	2.23	1.37–3.65	0.001
Positive	112 (64%)						
HER2 status							
Non-amplified	146 (83%)	1.07	0.62–1.83	0.82	0.87	0.46–1.67	0.67
Amplified	30 (17%)						
Grade							
3	120 (68%)	1.67	1.04–2.69	0.03	2.00	1.13–3.56	0.02
1-2	56 (32%)						
Stage							
I	44 (25%)	1.03	0.61–1.73	0.91	1.71	0.85–3.46	0.13
IIA/IIB	118 (67%)						
IIIA/IIIB	14 (8%)						
Gene expression microarray score for TIMP-1							
Range	−5.80–4.38	1.64	1.02–2.61	0.04	1.29	0.74–2.23	0.37
≥0.84	38 (21.6%)						
<0.84	138 (78.4%)						

*P* values correspond to univariate Cox regression analysis. HR hazard ratio.

**Table 2:** Occurrence of clinical/pathological features in patients with relative TIMP-1 RNA levels of ≥0.84 (*n* = 38) compared to patients with relative TIMP-1 RNA levels of <0.84 (*n* = 138).

TIMP-1 RNA	≥0.84	<0.84	*P*
Total	38	138	
Recurrence	24	67	0.11
Death	17	51	0.38
Negative ER status	15	49	0.65
High grade	29	91	0.22
HER2 amplified	13	17	0.001
Stage I	6	38	
Stage IIA/IIB	27	91	0.18
Stage IIIA/IIIB	5	9	

**Table 3:** Multivariate analysis for recurrence-free survival using TIMP-1 mRNA levels.

Parameter	Hazard ratio	95% CI	*P* value
TIMP-1 mRNA levels	1.68	1.02–2.78	0.04
ER status	1.32	0.81–2.15	0.27
HER2 status	0.90	0.50–1.59	0.71
Stage	0.95	0.55–1.63	0.84
Grade	1.56	0.91–2.68	0.11

**Table 4:** Clinicopathologic features of the patients included in the TMA analysis.

		Univariate analysis			
		Recurrence		Death	
		HR	*P*	HR	*P*
Number of patients	145				
Age at diagnosis					
Median	52 years				
Range	26–89 years				
Recurrence					
Events	78 (54%)				
Death					
Events	61 (42%)				
ER status					
Positive	90 (62%)	1.41 (0.89–2.24)	0.14	1.89 (1.13–3.14)	0.01
Negative	55 (48%)				
HER2 status					
Amplified	25 (18%)	0.99 (0.56–1.77)	0.98	0.7 (0.34–1.42)	0.32
Non-amplified	117 (82%)				
Grade					
3	103 (71%)	1.39 (0.83–2.33)	0.22	1.52 (0.84–2.77)	0.17
1-2	42 (29%)				
Stage					
I	31 (21%)	0.99 (0.55–1.80)	0.98	1.34 (0.64–2.83)	0.44
IIA/IIB	102 (70%)				
IIIA/IIIB	12 (9%)				
Cytoplasmic TIMP-1 score					
Low (0, 1+, 2+)	51 (35%)	1.02 (0.65–1.62)	0.92	1.41 (0.83–2.42)	0.21
High (3+)	94 (65%)				

**Table 5:** Occurrence of clinical/pathological features in patients with high levels of TIMP-1 protein (*n* = 94) compared to patients with low levels of TIMP-1 protein (*n* = 51).

Cytoplasmic TIMP-1 score	High (3+)	Low (0, 1+, 2+)	*P*
Total	94	51	
Recurrence	49	29	0.59
Death	41	20	0.61
Negative ER status	40	15	0.12
High grade	71	32	0.11
HER2 amplified	18	7	0.36
Stage I	18	13	
Stage IIA/IIB	66	36	0.30
Stage IIIA/IIIB	10	2	

**Table 6:** Articles studied the association between TIMP-1 and prognosis.

Study	Specimen type	Molecule studied	Methodology	Sample size (*N*)	End points	Results	*P* value
Schrohl et al. 2004 [10]	Tumor tissue cytosolic extracts	Protein	ELISA	2984	Recurrence-free and overall survival	HR 1.37 HR 1.21 (high versus low levels)	*P* < 0.001 *P* = 0.003
Wu et al. 2008 [6]	Serum and paraffin-embedded tumor tissue	Protein and mRNA	ELISA, IHC and *in situ *hybridization	60	Recurrence-free and overall survival rate	50% versus 75% 55% versus 88% (high versus low levels)	*P* = 0.082 *P* = 0.03
Nakopoulou et al. 2002 [19]	Paraffin-embedded tumor tissue	mRNA	*In situ *hybridization	117	Overall survival	HR 1.53 (high versus low levels)	*P* = 0.042
Nakopoulou et al. 2003 [15]	Paraffin-embedded tumor tissue	Protein	IHC	133	Disease-free survival	TIMP-1 overexpression was a favorable prognostic factor.	*P* = 0.01
Sieuwerts et al. 2007 [16]	Frozen tumor tissue	mRNA	RT-PCR	1301	Metastasis-free survival	HR 0.59 (high versus low levels)	*P* < 0.001
Current study	Paraffin-embedded tumor tissue	Protein	IHC	176	Recurrence-free and overall survival	HR 1.2 HR 1.73 (high versus low levels)	*P* = 0.35 *P* = 0.03
	Snap-frozen tumor tissue	mRNA	Tissue microarray	176	Recurrence-free and overall survival	HR 1.6 HR 1.29 (high versus low levels)	*P* = 0.04 *P* = 0.37

## References

[1] Porter P (2008). “Westernizing” women’s risks? Breast cancer in lower-income countries. *New England Journal of Medicine*.

[3] Rakha EA, Reis-Filho JS, Baehner F (2010). Breast cancer prognostic classification in the molecular era: the role of histological grade. *Breast Cancer Research*.

[4] Würtz SØ, Schrohl AS, Møller Sørensen N (2005). Tissue inhibitor of metalloproteinases-1 in breast cancer. *Endocrine-Related Cancer*.

[5] Würtz SØ, Schrohl AS, Mouridsen H, Brünner N (2008). TIMP-1 as a tumor marker in breast cancer—an update. *Acta Oncologica*.

[6] Wu ZS, Wu Q, Yang JH (2008). Prognostic significance of MMP-9 and TIMP-1 serum and tissue expression in breast cancer. *International Journal of Cancer*.

[7] McCarthy K, Maguire T, McGreal G (1999). High levels of tissue inhibitor of metalloproteinase-1 predict poor outcome in patients with breast cancer. *International Journal of Cancer*.

[8] Kuvaja P, Würtz SØ, Talvensaari-Mattila A, Brünner N, Pääkkö P, Turpeenniemi-Hujanen T (2007). High serum TIMP-1 correlates with poor prognosis in breast carcinoma—a validation study. *Cancer Biomarkers*.

[9] Schrohl AS, Christensen IJ, Pedersen AN (2003). Tumor tissue concentrations of the proteinase inhibitors tissue inhibitor of metalloproteinases-1 (TIMP-1) and plasminogen activator inhibitor type 1 (PAI-1) are complementary in determining prognosis in primary breast cancer. *Molecular & Cellular Proteomics*.

[10] Schrohl AS, Holten-Andersen MN, Peters HA (2004). Tumor tissue levels of tissue inhibitor of metalloproteinase-1 as a prognostic marker in primary breast cancer. *Clinical Cancer Research*.

[11] Talvensaari-Mattila A, Turpeenniemi-Hujanen T (2005). High preoperative serum TIMP-1 is a prognostic indicator for survival in breast carcinoma. *Breast Cancer Research and Treatment*.

[12] Würtz SØ, Møller S, Mouridsen H, Hertel PB, Friis E, Brünner N (2008). Plasma and serum levels of tissue inhibitor of metalloproteinases-1 are associated with prognosis in node-negative breast cancer: a prospective study. *Molecular and Cellular Proteomics*.

[13] Schrohl AS, Meijer-Van Gelder ME, Holten-Andersen MN (2006). Primary tumor levels of tissue inhibitor of metalloproteinases-1 are predictive of resistance to chemotherapy in patients with metastatic breast cancer. *Clinical Cancer Research*.

[14] Lipton A, Leitzel K, Chaudri-Ross HA (2008). Serum TIMP-1 and response to the aromatase inhibitor letrozole versus tamoxifen in metastatic breast cancer. *Journal of Clinical Oncology*.

[15] Nakopoulou L, Giannopoulou I, Lazaris AC (2003). The favorable prognostic impact of tissue inhibitor of matrix metalloproteinases-1 protein overexpression in breast cancer cells. *Acta Pathologica, Microbiologica, et Immunologica Scandinavica*.

[16] Sieuwerts AM, Usher PA, Meijer-Van Gelder ME (2007). Concentrations of TIMP1 mRNA splice variants and TIMP-1 protein are differentially associated with prognosis in primary breast cancer. *Clinical Chemistry*.

[17] Germain DR, Graham K, Glubrecht DD, Hugh JC, MacKey JR, Godbout R (2011). DEAD box 1: a novel and independent prognostic marker for early recurrence in breast cancer. *Breast Cancer Research and Treatment*.

[18] Liu RZ, Graham K, Glubrecht DD, Germain DR, Mackey JR, Godbout R (2011). Association of FABP5 expression with poor survival in triple-negative breast cancer: implication for retinoic acid therapy. *American Journal of Pathology*.

[19] Nakopoulou L, Giannopoulou I, Stefanaki K (2002). Enhanced mRNA expression of tissue inhibitor of metalloproteinase-1 (TIMP-1) in breast carcinomas is correlated with adverse prognosis. *Journal of Pathology*.

[20] Willemoe GL, Hertel PB, Bartels A (2009). Lack of TIMP-1 tumour cell immunoreactivity predicts effect of adjuvant anthracycline-based chemotherapy in patients (*n* = 647) with primary breast cancer. A Danish Breast Cancer Cooperative Group Study. *European Journal of Cancer*.

[21] Ejlertsen B, Jensen MB, Nielsen KV (2010). HER2, TOP2A, and TIMP-1 and responsiveness to adjuvant anthracycline- containing chemotherapy in high-risk breast cancer patients. *Journal of Clinical Oncology*.

[22] Ejlertsen B, Jensen MB, Nielsen KV (2010). HER2, TOP2A, and TIMP-1 and responsiveness to adjuvant anthracycline- containing chemotherapy in high-risk breast cancer patients. *Journal of Clinical Oncology*.

[23] Fong KM, Kida Y, Zimmerman PV, Smith PJ (1996). TIMP1 and adverse prognosis in non-small cell lung cancer. *Clinical Cancer Research*.

[24] Gouyer V, Conti M, Devos P (2005). Tissue inhibitor of metalloproteinase 1 is an independent predictor of prognosis in patients with nonsmall cell lung carcinoma who undergo resection with curative intent. *Cancer*.

[25] Yukawa N, Yoshikawa T, Akaike M (2004). Prognostic impact of tissue inhibitor of matrix metalloproteinase-1 in plasma of patients with colorectal cancer. *Anticancer Research*.

[26] Yukawa N, Yoshikawa T, Akaike M (2008). Impact of plasma tissue inhibitor of matrix metalloproteinase-1 on long-term survival in patients with colorectal cancer. *Oncology*.

[27] Møller Sørensen N, Vejgaard Sørensen I, Ørnbjerg Wurtz S (2008). Biology and potential clinical implications of tissue inhibitor of metalloproteinases-1 in colorectal cancer treatment. *Scandinavian Journal of Gastroenterology*.

[28] Yoshikawa T, Tsuburaya A, Kobayashi O (2000). Prognostic value of tissue inhibitor of matrix metalloproteinase-1 in plasma of patients with gastric cancer. *Cancer Letters*.

[29] Yoshikawa T, Cho H, Tsuburaya A, Kobayashi O (2009). Impact of plasma tissue inhibitor of metalloproteinase-1 on long-term survival in patients with gastric cancer. *Gastric Cancer*.

[30] Scrideli CA, Cortez MAA, Yunes JA (2010). mRNA expression of matrix metalloproteinases (MMPs) 2 and 9 and tissue inhibitor of matrix metalloproteinases (TIMPs) 1 and 2 in childhood acute lymphoblastic leukemia: potential role of TIMP1 as an adverse prognostic factor. *Leukemia Research*.

[31] Jiang Y, Goldberg ID, Shi YE (2002). Complex roles of tissue inhibitors of metalloproteinases in cancer. *Oncogene*.

[32] Yamashita K, Suzuki M, Iwata H (1996). Tyrosine phosphorylation is crucial for growth signaling by tissue inhibitors of metalloproteinases (TIMP-1 and TIMP-2). *FEBS Letters*.

[33] Wang T, Yamashita K, Iwata K, Hayakawa T (2002). Both tissue inhibitors of metalloproteinases-1 (TIMP-1) and TIMP-2 activate Ras but through different pathways. *Biochemical and Biophysical Research Communications*.

[34] Jung KK, Liu XW, Chirco R, Fridman R, Kim HRC (2006). Identification of CD63 as a tissue inhibitor of metalloproteinase-1 interacting cell surface protein. *EMBO Journal*.

[35] Stetler-Stevenson WG (2008). Tissue inhibitors of metalloproteinases in cell signaling: metalloproteinase-independent biological activities. *Science Signaling*.

[36] Li H, Nishio K, Yamashita K, Hayakawa T, Hoshino T (1995). Cell cycle-dependent localization of tissue inhibitor of metalloproteinases-1 immunoreactivity in cultured human gingival fibroblasts. *Nagoya Journal of Medical Science*.

[37] Kuvaja P, Hulkkonen S, Pasanen I (2012). Tumor tissue inhibitor of metalloproteinases-1 (TIMP-1) in hormone-independent breast cancer might originate in stromal cells, and improves stratification of prognosis together with nodal status. *Experimental Cell Research*.

